# Non-respiratory particles emitted by guinea pigs in airborne disease transmission experiments

**DOI:** 10.1038/s41598-021-96678-w

**Published:** 2021-09-01

**Authors:** Sima Asadi, Manilyn J. Tupas, Ramya S. Barre, Anthony S. Wexler, Nicole M. Bouvier, William D. Ristenpart

**Affiliations:** 1 Department of Chemical Engineering, University of California Davis, One Shields Ave., Davis, CA 95616 USA; 2 Department of Microbiology, Icahn School of Medicine at Mount Sinai, 1 Gustave L. Levy Place, New York, NY 10029 USA; 3 Department of Mechanical and Aerospace Engineering, University of California Davis, One Shields Ave., Davis, CA 95616 USA; 4 Air Quality Research Center, University of California Davis, One Shields Ave., Davis, CA 95616 USA; 5 Department of Civil and Environmental Engineering, University of California Davis, One Shields Ave., Davis, CA 95616 USA; 6 Department of Land, Air and Water Resources, University of California Davis, One Shields Ave., Davis, CA 95616 USA; 7 Department of Medicine, Div. of Infectious Diseases, Icahn School of Medicine at Mount Sinai, 1 Gustave L. Levy Place, New York, NY 10029 USA; 8 Present Address: Department of Chemical Engineering, Massachusetts Institute of Technology, 77 Massachusetts Ave., Cambridge, MA 02139 USA; 9 Present Address: Department of Ecology and Evolutionary Biology, 304 Guyot Hall, Princeton University, Princeton, NJ 08544 USA

**Keywords:** Influenza virus, Biomedical engineering

## Abstract

Animal models are often used to assess the airborne transmissibility of various pathogens, which are typically assumed to be carried by expiratory droplets emitted directly from the respiratory tract of the infected animal. We recently established that influenza virus is also transmissible via “aerosolized fomites,” micron-scale dust particulates released from virus-contaminated surfaces (Asadi et al. in Nat Commun 11(1):4062, 2020). Here we expand on this observation, by counting and characterizing the particles emitted from guinea pig cages using an Aerodynamic Particle Sizer (APS) and an Interferometric Mie Imaging (IMI) system. Of over 9000 airborne particles emitted from guinea pig cages and directly imaged with IMI, none had an interference pattern indicative of a liquid droplet. Separate measurements of the particle count using the APS indicate that particle concentrations spike upwards immediately following animal motion, then decay exponentially with a time constant commensurate with the air exchange rate in the cage. Taken together, the results presented here raise the possibility that a non-negligible fraction of airborne influenza transmission events between guinea pigs occurs via aerosolized fomites rather than respiratory droplets, though the relative frequencies of these two routes have yet to be definitively determined.

## Introduction

The physical pathways governing airborne disease transmission remain poorly understood, in part due to the lack of quantitative data on the number of pathogens emitted by infected individuals and then inhaled by susceptible individuals^1–4^. Because of the difficulty of performing controlled infectious disease transmission experiments with human subjects, animal models have been instead widely used to perform transmission experiments with influenza viruses^5–14^, and less often with other microorganisms that can travel through the air to infect the respiratory tract of a susceptible host, including *Mycobacterium tuberculosis*^15^, Ebola virus^16^, and SARS-CoV-2^17,18^. Typically, an animal inoculated with the pathogen is placed in a separate cage in the vicinity of a naïve, uninfected partner animal such that air can circulate freely through both cages, but direct and indirect contact between the partner animals is precluded. If the naïve animal becomes infected, the pathogen is considered to be transmissible by an airborne route.

The usual implicit assumption with respiratory viruses like influenza virus or SARS-CoV-2 is that respiratory emissions from the infected animal carry the pathogen to the susceptible animal. Many authors explicitly refer to the airborne pathogen transporters as “respiratory droplets”^9,11,19–21^; however, in most indoor human environments, water-laden droplets in the micron-scale range rapidly evaporate within seconds after exhalation to become “droplet nuclei,” the residual, non-volatile proteins, lipids, and salts in respiratory fluid^22,23^. It is these droplet nuclei, sometimes also called “aerosols,” which may remain airborne for long periods of time before eventually being inhaled into the respiratory tract of a susceptible host. The relative frequency of transmission via large respiratory droplets versus smaller droplet nuclei remains unknown. However, the conventional experimental setup used to test airborne virus transmission in animal models does not conclusively prove that the virus infecting the susceptible animal was directly exhaled into the air by the inoculated animal. It establishes that a pathogen is airborne-transmissible, but does not establish the composition or origin of the airborne particles transmitting the pathogen.

Recent work by our team in the guinea pig model^24^ established that influenza is transmissible through the air on “aerosolized fomites,” the name given to microscopic particles emitted from virus-contaminated surfaces such as skin, fur, or bedding. We used an aerodynamic particle sizer (APS) and video recordings to establish quantitatively that the vast majority of particles emitted from a guinea pig cage are environmental dust, not respiratory droplets. We showed that, even in the absence of a donor animal with active respiratory tract infection, influenza virus in the environment could become airborne and transmit to a susceptible recipient guinea pig nearby, and also that aerosolized fomites could be readily generated from friable materials like virus-contaminated facial tissues. These observations strongly suggest that some unknown fraction of transmission events observed in the guinea pig model are due to aerosolized fomites, rather than expiratory droplets, as is commonly assumed.

In this work we provide more details about the nature and origin of the airborne particles emanating from guinea pig cages, focusing on the physical characteristics of the particles. We designed an experimental setup based on interferometric Mie imaging (IMI)^25–29^ to quantify the number of aerosol particles emitted from guinea pig cages and to qualitatively characterize these particles by examining the interference patterns made by light waves reflected and refracted by the particles as they pass through a laser sheet^30^. With IMI, spherical, homogenous, and transparent particles, including liquid droplets, yield regular fringes in the out-of-focus wave-interference patterns captured by a camera as the laser sheet illuminates the droplets^27,28^. Nontransparent solid, partially liquid, or heterogeneous objects, in contrast, yield complicated speckle patterns with no clear fringe spacing^31^, allowing IMI to discriminate between liquid droplets and solid or mixed particulates in the air. We also used an Aerodynamic Particle Sizer (APS) to measure the number and size distribution of airborne particles sampled from guinea pig cages under various experimental conditions. Preliminary results with the APS were reported previously^24^; here we add an independent measuring technique, IMI, to compare with APS data and expand upon our analyses of the dynamics of airborne particle generation by awake and anesthetized guinea pigs used in modeling the airborne transmission of influenza viruses.

## Experimental methods

### IMI setup

**Figure 1:**
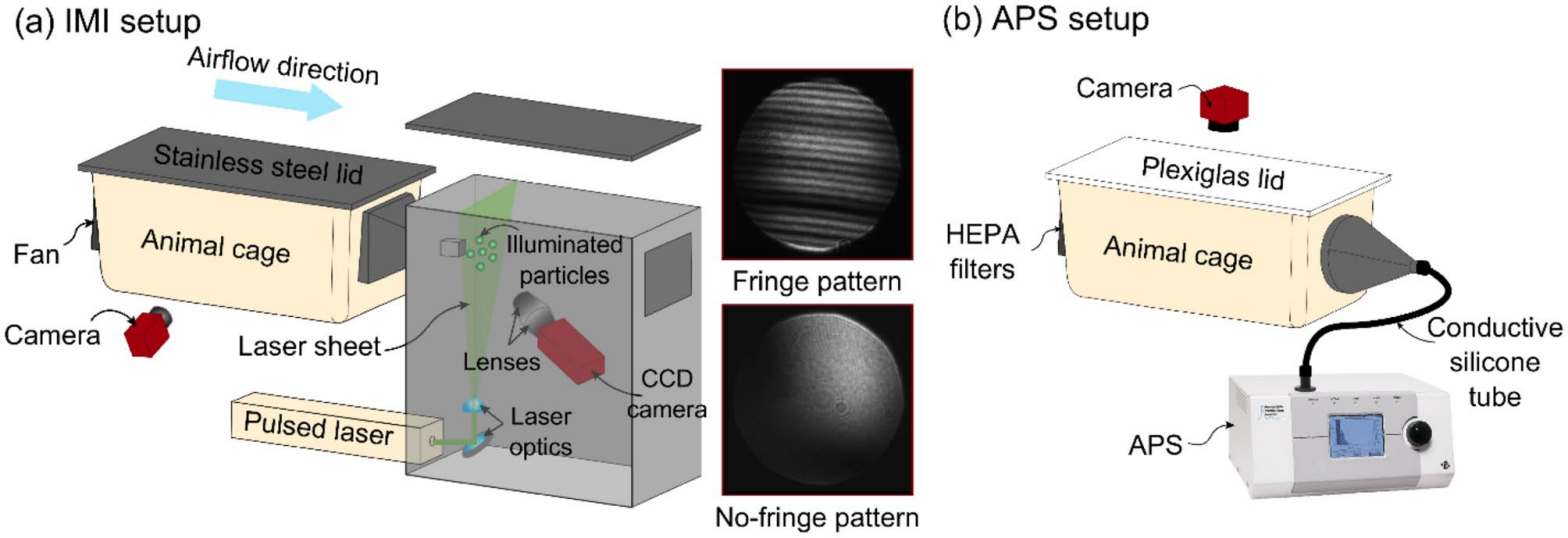
Schematic of interferometric Mie imaging (IMI) and aerodynamic particle sizer (APS) setup. (**a**) IMI setup for counting and qualitatively characterizing particles emitted from a guinea pig cage. Particles emitted from the cage are carried toward the laser sheet by a horizontal airflow. A CCD camera captures out-of-focus images of the illuminated particles; images at right show representative water droplets with diameter of > 2 µm (fringe pattern), and < 2 µm (no-fringe pattern). A second camera captures the time-lapse images of guinea pig in the cage (side-view). (**b**) APS setup for measuring the size distribution of particles with diameter of 0.3–20 µm emitted from a guinea pig cage. An APS pulls the air at 5 L/min through the HEPA filters and a camera captures top-view images of the guinea pig in the cage at 1 image per second.

An experimental apparatus was designed to count and qualitatively characterize the aerosol particles emitted from an animal cage using IMI (Fig. 1a and Fig. S1a). This technique involves the capture of out-of-focus images of the particles illuminated in a laser sheet. Interference between the light reflected by and refracted in a transparent sphere, such as a water droplet, yields a pattern of evenly spaced fringes, the width of which is inversely proportional to the droplet size^26,29^. Our experimental apparatus consisted of a standard animal cage (26.7 cm × 48.3 cm × 20.3 cm) connected to an enclosed black box (22 cm × 44 cm × 46 cm) containing the optical equipment. A 532 nm single-pulsed laser beam (Nd:YAG Nano L 20–290 Litron, max pulse rate = 20 Hz, pulse length = 1 ns) was directed inside the box and converted to a laser sheet (5 mm thickness) with a mirror and laser line generator. During the experiment, a fan mounted inside the guinea pig cage pushed airborne particles toward the laser sheet through a 2.54 cm × 2.54 cm hole. A charge-coupled device (CCD) camera (Allied Vision Prosilica) and two camera lenses (Zeiss Planar T*85 mm f/1.4) captured the out-of-focus images of the particles illuminated in the laser sheet. The lenses were attached together at the front of the camera, capturing an area of 8.7 mm × 7 mm and enabling measurement of particle size down to 2 μm in diameter^29^. Because the aperture was approximately 4 times larger than this field of view, our setup did not measure 100% of the particles, and the particle emission rates reported here do not represent the absolute number of particles emitted from the cage. For laser safety reasons, and also to minimize background light contamination, a light-absorbing blackout curtain was wrapped around the guinea pig cage (not shown). A second webcam camera (Logitech HD webcam C310) was placed inside the curtain to capture side view images of the guinea pig inside the cage at 1 image per second, and standard image analysis techniques were used to track the motion of the guinea pig^24^. Red LED lights illuminated the guinea pigs without interfering with acquisition of particle images. Fig. S1b and Fig. S1c show representative image analyses of detected guinea pigs (green lines) and their corresponding centroids (red circles). Guinea pig movement velocity was calculated by measuring the change in position of the centroid between consecutive time-lapse photographs. Simultaneously, the CCD camera inside the black box recorded out-of-focus images of particles illuminated by laser sheet at an acquisition rate of 10 images per second.

Before starting the experiment, the setup was tested with deionized water droplets generated using a humidifier (AGPtek Mini USB air mist humidifier) and also droplets emitted from human speech released approximately 3 cm away from the laser sheet, in a manner similar to that reported previously^30^. In both cases, we observed two interference patterns (Fig. 1a): regularly spaced light fringes, indicating transparent droplets larger than 2 µm in diameter, or a single, uniformly bright fringe in images of droplets smaller than 2 µm.

The following experiments were performed:

(i) An uninfected, unrestrained guinea pig on dried corncob (CC) bedding was placed inside the measurement cage, and cage air was sampled for 60 min (Fig. S1b). Before placing the guinea pig in the cage, background particles were measured for 30 min (No GP (background)).
(ii) The CC bedding was replaced with bedding made of polar fleece (PF)-covered absorbent pads (Fisherbrand Universal All-Purpose Absorbent Pads, Fisher Healthcare). All other conditions were similar to the CC bedding experiment (Fig. S1c).
(iii) An anesthetized, uninfected guinea pig was used to characterize the airborne particles produced by tidal breathing. After anesthetizing the guinea pig, it was placed inside a plastic bag to prevent the aerosolization of dander and fur (Fig. S1d), and the expiratory particles were collected through a small hole made for the guinea pig’s nose (Fig. S1e). The plastic bag containing the anesthetized guinea pig was placed in the empty cage on a platform about 8 cm away from the laser sheet with its nose facing toward the laser sheet. Before placing the anesthetized guinea pig, the empty cage background particles were measured for 60 min.

Food and water were provided for awake guinea pigs during the measurements; for anesthetized guinea pigs, food, water, and bedding were removed from the cage. Each experiment was repeated three times each for three guinea pigs, denoted as GP1, GP2, and GP3. To compare the particle emission rates for different conditions in a manner consistent with our prior work^24^, we time-averaged the particle emission rates over 15-min periods, denoted as ${\overline{N} }_{(15)}$. The particle emission rates reported for awake guinea pigs are the average of four 15-min periods, each acquired over an hour, with three individual hour-long trials per guinea pig, yielding a total sample size of $4\times 3\times 3=36$ measurements per experimental condition. To test for correlation between particle emission and animal motion velocity, we time-averaged the particle emission rate (${\overline{N}}_{(1)}$) and guinea pig movement velocity (${\overline{V}}_{(1)}$) over 1-min periods.

### APS setup

Preliminary results using the APS were reported previously^24^; here we provide more details about the dynamics of particle emission and corresponding particle size distributions for trial replicates under various conditions. In brief, the setup consisted of an airtight standard animal cage (26.7 cm × 48.3 cm × 20.3 cm) with a Plexiglas lid connected to an aerodynamic particle sizer (APS, TSI model 3321) through a static dissipative silicon tube (Fig. 1b, Fig. S2). The setup was placed inside a biosafety cabinet (NUAIRE, NU-430 Class II Type B2) to minimize the background particle concentration. The APS pulls the air at a total flow rate of 5 L/min (sheath flow rate ≅ 4 L/min, sample flow rate ≅ 1 L/min) through two HEPA filters attached to the cage, and records the number and size of particles between 0.5 and 20 µm in diameter. It also counts but cannot size particles between 0.37 and 0.5 μm, due to limits in resolution. The APS acquisition time was set to report the cumulative number of particles detected per second. A camera positioned above the cage captured time-lapse photographs at 1 image per second, and guinea pig movement velocity was calculated as described above. The following experiments with different types of bedding (see Fig. S1 of Asadi et al.^24^) were performed:

(i) Similar to the IMI setup experiments, an unrestrained, uninfected guinea pig was placed in the measurement cage on CC bedding during a 60-min air sampling. Cage background particle count was also measured for 60 min before placing the guinea pig in the cage (No GP (background)).
(ii) CC bedding was replaced with PF bedding, and all other conditions were similar to the CC bedding experiment.
(iii) The bedding was removed from the cage (No bedding), and all other conditions were similar to the CC and PF bedding experiments.
(iv) To measure the expiratory particles released from a guinea pig inoculated with influenza A virus, an anesthetized animal was placed inside an aluminum sleeve (20 cm × 10 cm × 10 cm) with a small hole (3.5 cm in diameter) for its nose (see Fig. S4 of Asadi et al.^24^), to minimize background particles such as fur or dander. The aluminum sleeve replaced the plastic bag used in the IMI setup to decrease any potential influence of static charge. The aluminum sleeve was then placed inside the measurement cage with the guinea pig’s nose directly facing a stainless-steel funnel attached to the APS inlet. The measurements were performed at 0, 1, 2, and 3 days post-inoculation (dpi); on 0 dpi, the measurements were taken before virus inoculation.
(v) As a negative control, the experiment described above (iv) was repeated after each guinea pig was humanely euthanized and then placed inside the aluminum sleeve.

Food and water were provided for unrestrained, awake guinea pigs during measurements; with anesthetized or euthanized guinea pigs, no food, water or bedding was present in the cage. Each experiment with APS setup was performed once with three different guinea pigs denoted as GP4, GP5, and GP6.

### Virology and animals

All procedures were performed in strict accordance with the recommendations in the Guide for the Care and Use of Laboratory Animals, and the research protocol was approved by the Icahn School of Medicine at Mount Sinai Institutional Animal Care and Use Committee (IACUC protocol #2014-0178). Reporting in this manuscript follows recommendations in the ARRIVE guidelines. All methods for viruses, plaque assays, and guinea pigs used for the experiments reported here are identical to our previous work^24^; interested readers are referred there for details about the virology and transmission rate results. The primary focus here is on the characterization of the aerosol particles emitted from the cage.

## Results

### Observation of speckle-like pattern for particles emitted from an animal cage

Our preliminary experiments using both deionized water droplets generated by a humidifier, and respiratory droplets released from human speech, established that our IMI setup could successfully capture micron-scale liquid droplets (Fig. 1a).

**Figure 2:**
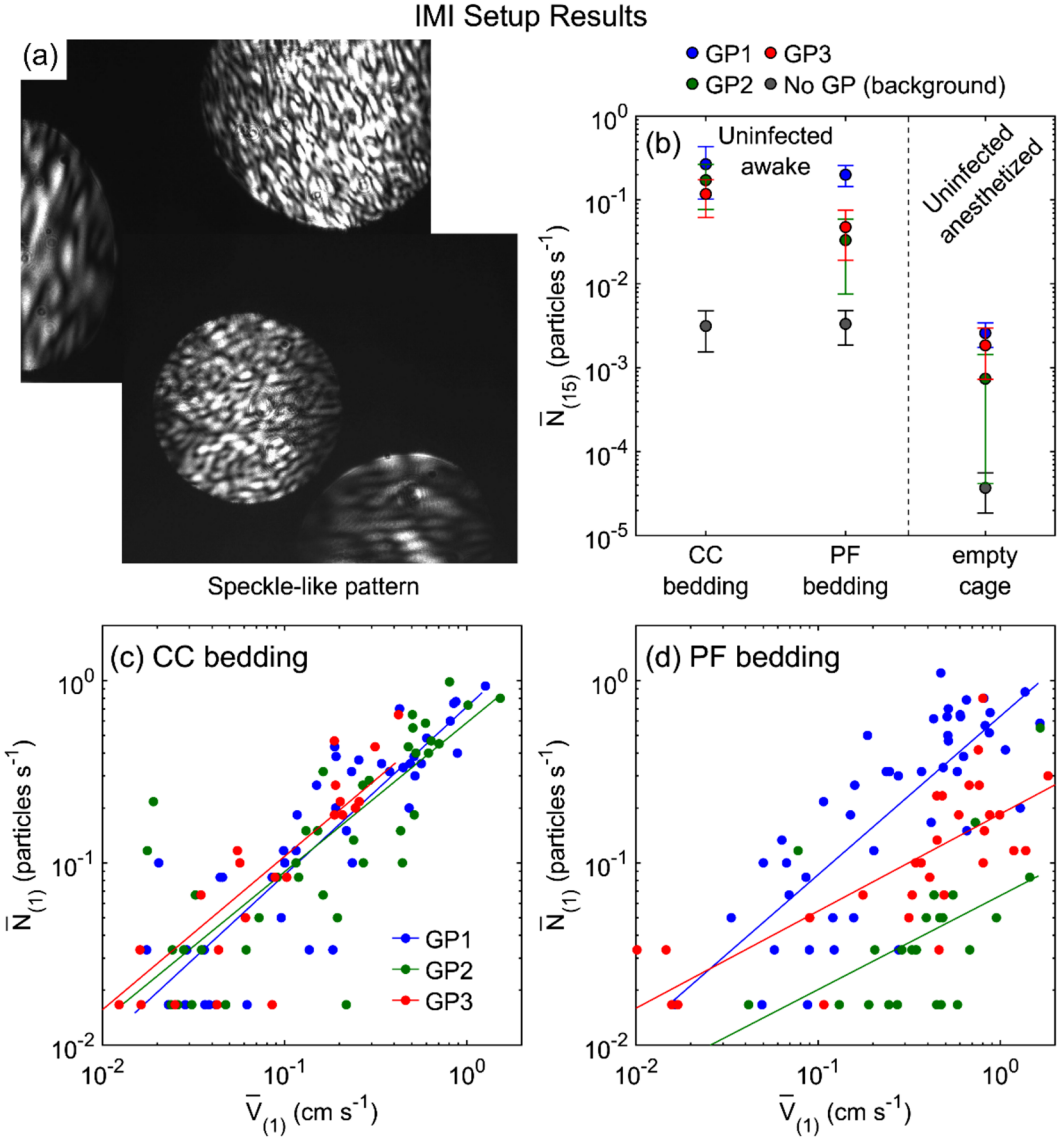
Particle emission rates measured by IMI setup. (**a**) Images captured by CCD camera that show speckle-like pattern for particles illuminated by the laser sheet. (**b**) Time-averaged particle emission rate, ${\overline{N} }_{\left(15\right)}$ for three guinea pigs (GP1, GP2, and GP3) under different conditions: uninfected, awake guinea pig in the cage with CC bedding or PF bedding, and uninfected, anesthetized guinea pig inside the empty cage. A plastic bag was used to cover anesthetized guinea pig fur with a small opening for its nose facing directly toward the laser sheet. Background particle measurements for each condition where no guinea pig was in the cage are shown with gray circle markers (No GP (background)). Each data point is the average of four 15-min time-averaged particle emission rates and 3 trials for each guinea pig. Time-averaged particle emission rate, ${\overline{N} }_{\left(1\right)}$ versus time-averaged guinea pig movement velocity, ${\overline{V} }_{\left(1\right)}$ over 1 min, for three uninfected guinea pigs in the cage with (**c**) CC bedding, and (d) PF bedding. Solid lines are best power law fits (see Table S1 for statistics).

Over a cumulative total of 18 h of observation time with 3 awake, unrestrained guinea pigs, our IMI system recorded a total of 9046 airborne particles emanating from the measurement cage, all of which had speckled patterns (Fig. 2a), consistent with particulates that either are non- spherical or have non-homogenous index of refraction. Likewise, for the anesthetized, stationary animals, over a cumulative total of 9 h of observation time with three different animals, we observed a total of 56 objects, all of which again had speckled patterns. We did not observe any airborne particles with clear fringes, which would be characteristic of a liquid droplet > 2 μm, nor any that were uniformly bright, suggesting a droplet < 2 μm. In other words, we obtained no evidence of airborne droplets emerging from the cages of guinea pigs that were either awake and mobile or anesthetized and stationary.

Although no clearly liquid droplets were observed, it is possible that some fraction of the particulates were the dried respiratory particles called droplet nuclei. The evaporation time scale of micron-scale water droplets is approximately 100 ms for a 1-micron droplet to several seconds for a 20-micron droplet for our experimental temperature and humidity range^32,33^, while the time required for a particle to reach the laser sheet from the nose of the guinea pig in our setup is at least 1 s. This comparison suggests that any micron-scale expiratory droplets or droplets from other sources might have fully dried prior to reaching the laser sheet, generating nonhomogeneous droplet nuclei or dry particles, and this heterogeneity leads to deviation from Mie scattering and disappearance of the fringes^31^. We emphasize that we are not able to differentiate solid dust particulates from dried expiratory droplets based on the scattering patterns.

Although the speckle pattern does not allow any quantitative information to be extracted about the size of particles, other evidence suggests that the particles are primarily environmental dust. First, despite the lack of quantitative fringe measurement, information can still be obtained about the absolute number of solid particulates emitted from the cage. Figure 2b shows the time-averaged particle emission rate over 15 min, ${\overline{N} }_{(15)}$, for three different experimental conditions examined with the IMI setup. We first quantified the particles emitted from a cage while the guinea pig was awake and mobile either on CC or on PF bedding. We reiterate that the CCD camera field of view was only 8.7 mm × 7 mm, so this setup does not necessarily capture all of the emitted particles. Nonetheless, the IMI data indicate that one to two orders of magnitude more particulates were emitted from the cage by awake mobile guinea pigs in comparison to the background level, with slightly more particulates emitted from guinea pigs in cages with CC bedding than PF bedding. Next, to measure the number of expiratory particles and minimize the background fur and dander particulates, we anesthetized the guinea pigs and placed them individually in a plastic bag with a small opening for the guinea pig nose. The results (Fig. 2b) demonstrated that ${\overline{N} }_{(15)}$ for anesthetized guinea pigs (~ 0.002 particles/s) is two orders of magnitude lower than awake mobile guinea pigs (~ 0.14 particles/s). There was only a small difference between the particle emission rates for the anesthetized guinea pig when mostly covered in plastic and the background particles for a cage with the bedding but no guinea pig.

Post-processing of the images captured to track guinea pig movement in the cage allowed calculation of the guinea pig velocity within the cage. The time-averaged particle emission rate versus time-averaged guinea pig velocity over 1 min (${\overline{N} }_{(1)}$ vs. ${\overline{V} }_{(1)}$) is plotted in Fig. 2b, c, which show that the particle emission rate as measured by IMI is correlated with guinea pig movement velocity for both CC bedding (Fig. 2c) and PF bedding (Fig. 2d) experiments. These results indicate that increased guinea pigs motion yielded more aerosolized particles, suggesting that the majority of particles emitted from a guinea pig cage are environmental dust or dander rather than expiratory particles.

### Observation of bimodal size distribution for particles emitted from an animal cage

**Figure 3:**
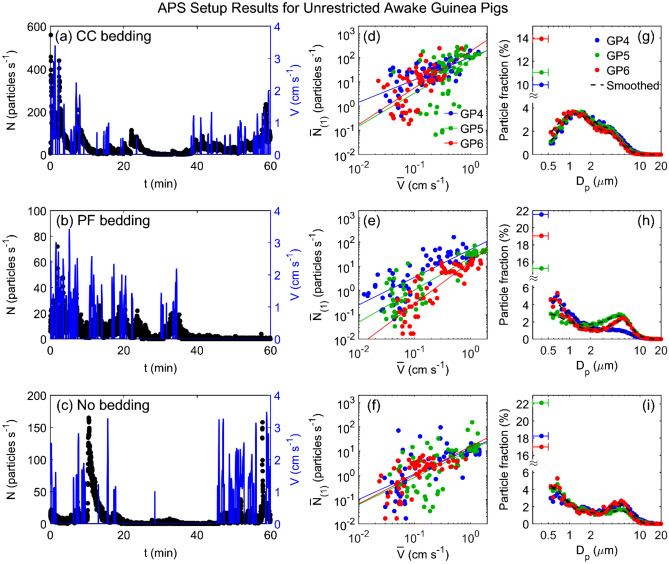
Particle emission rates measured by APS setup for unrestricted awake guinea pigs. Particle emission rate, $N$ (left axis) and guinea pig movement velocity, $V$ (right axis) versus time for a representative guinea pig in the cage with (**a**) CC bedding, (**b**) PF bedding, and (**c**) no bedding. Time-averaged particle emission rate, ${\overline{N} }_{\left(1\right)}$ versus time-averaged guinea pig movement velocity, ${\overline{V} }_{\left(1\right)}$, for three uninfected awake guinea pigs (GP4, GP5, and GP6) in the cage with (**d**) CC bedding, (**e**) PF bedding, and (**f**) no bedding. Solid lines are power law fits (see Table S2 for statistics). Corresponding size distributions (**g**, **h**, and **i**) for the uninfected awake guinea pigs (GP4, GP5, and GP6) in the cage with (**g**) CC bedding, (**h**) PF bedding, and (**i**) no bedding. The left-most data points in each plot shows the fraction of particles counted in the 0.3–0.5 μm bin, which cannot be further size-discriminated. The whiskers represent the width of the bin (0.3–0.5 μm). Dashed lines represent the data using a 5-point smoothing function. Data indicated by blue markers for GP4 in (**d**) and (**g**) are reproduced from Asadi et al.^24^ and included here for comparison.

Due to the lack of fringes in the observed (inhomogeneous and/or non-spherical) particulates, the IMI apparatus was unable to obtain any particle size measurements. Instead, we performed complementary experiments with the APS, which has increased spatial and temporal resolution for counting and sizing aerosol particles (albeit without yielding information about particle composition). Some APS data were reported previously^24^; here we provide more details regarding the size distributions, velocity correlations, and exponential decay behavior of the concentration spikes. Figure 3 shows representative measurements of the instantaneous particle emission rate, $N$, and concurrent guinea pig movement velocity, $V$, versus time, for an awake uninfected guinea pig placed in the measurement cage with CC bedding (Fig. 3a), PF bedding (Fig. 3b), or no bedding (Fig. 3c). These data clearly indicate that whenever the guinea pig moves, there is a spike in particle emission rate, and after the guinea pig ceases moving, the particle emission rate decreases gradually to the background level unless interrupted by another burst of motion. Notably, there is little evidence of the converse situation: we do not observe any appreciable spikes in the particle concentration without a preceding burst of animal motion. Scatter plots of the time-averaged particle emission rates (${\overline{N} }_{(1)}$) versus the time-averaged guinea pig velocity (${\overline{V} }_{(1)}$) yield positive correlations (Fig. 3d–f) similar to the trend observed with the IMI setup, for all three guinea pigs tested and all three bedding types. In other words, two completely separate measurement techniques yielded positive correlations between particle counts and animal motion.

The corresponding size distributions of particles emitted from the cage, measured in the range of 0.5–20 μm in diameter, are shown in Fig. 3g–i. The CC bedding measurements (Fig. 3g) exhibit a bimodal distribution, with count mean diameters at approximately 1 μm and 3.5 μm. For PF bedding (Fig. 3h) and no bedding (Fig. 3i) the bimodal distribution is preserved, although the first peak is shifted toward smaller sizes (approximately 0.5 μm), while the second peak is shifted toward larger sizes at approximately 5 μm. The size distribution results suggest that the particles emitted from the animal cage are generated from more than one source; the change in peak sizes with respect to the CC bedding (Fig. 3g) versus PF bedding (Fig. 3h) strongly indicates that particulates from the bedding itself comprises a substantial fraction of the aerosolized matter.

**Figure 4:**
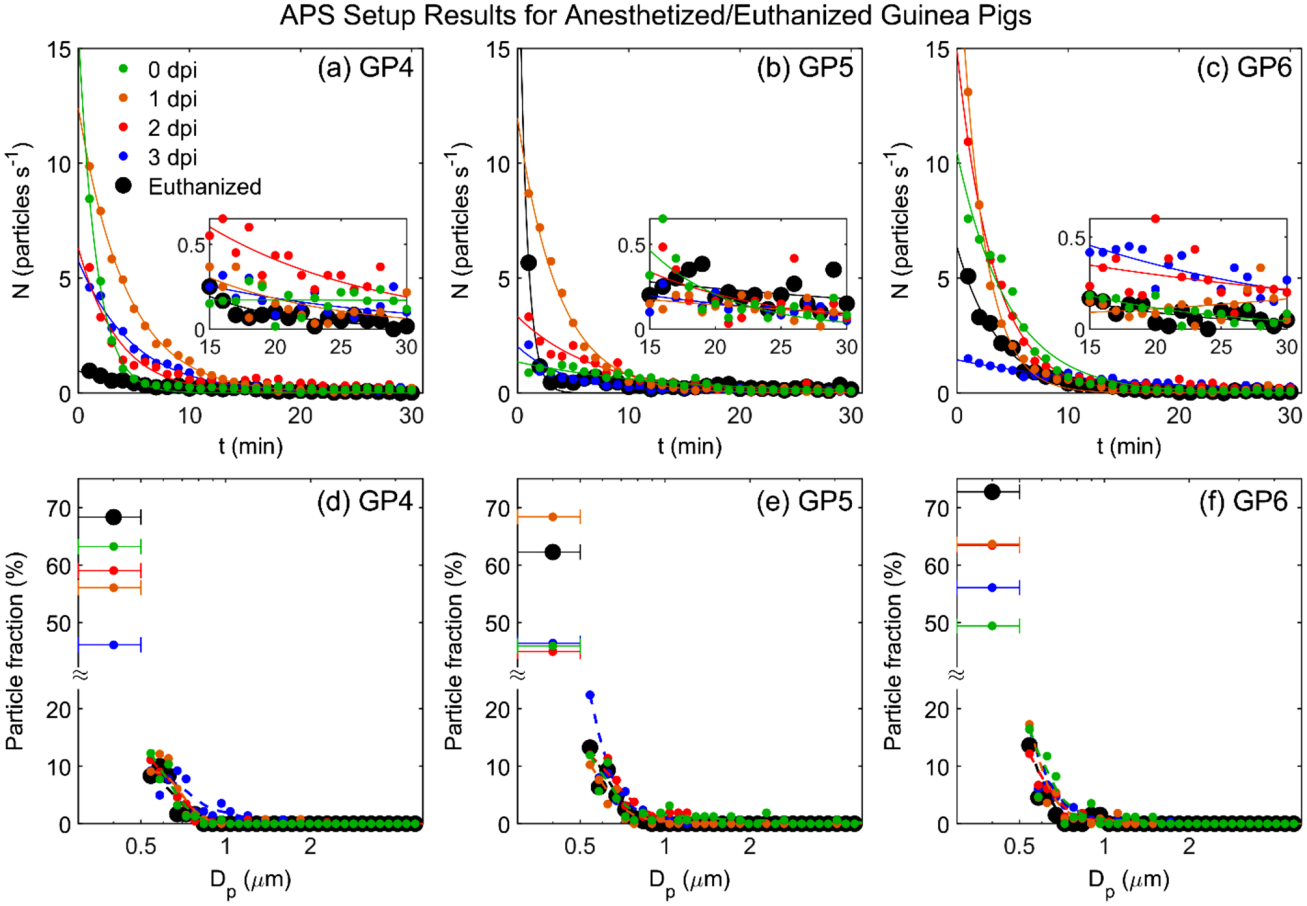
Particle emission from anesthetized or euthanized guinea pigs measured by the APS setup. Particle emission rate, $N$, versus time under 5 conditions: uninfected anesthetized guinea pig (0 dpi), inoculated anesthetized guinea pig at 1, 2, and 3 dpi, and euthanized guinea pig, for (**a**) GP4, (**b**) GP5, and (**c**) GP6. Magnifications show the final 15 min of each set. (**d**–**f**) Corresponding size distribution for particles emitted during the last 15 min of the measurements shown in (**a**)–(**c**). The left-most data points in each plot shows the fraction of particles counted in the 0.3–0.5 μm bin, which cannot be further size-discriminated. The whiskers represent the width of the bin (0.3–0.5 μm). Dashed lines represent the data using a 5-point smoothing function. Data indicated by red and black markers in (**d**) for GP4 at 2 dpi and euthanized, respectively, are reproduced from Asadi et al.^24^ and included here for comparison.

As with the IMI setup, we sought to minimize the dust and dander by anesthetizing and enclosing the guinea pigs to hopefully allow quantification of just the guinea pig expiratory particles. Anesthetized guinea pigs were placed individually in an aluminum sleeve with a small hole for its nose, and the sleeve was attached nose first to the funnel inside the measurement cage (see Fig. S4 of Asadi et al.^24^). Air necessarily flowed past the animal body in this configuration, but there was no motion besides the animal respiration. Figure 4a–c show the particle emission rate during 30 min of air sampling from uninfected anesthetized guinea pigs (0 dpi) and inoculated guinea pigs 1, 2 and 3 days post inoculation. Prior to placing the animal in the apparatus, the particle count was close to zero, i.e., the cage was ‘washed-out’ of particles. Shortly after placing the aluminum sleeve containing the anesthetized guinea pig inside the measurement cage, the number of emitted particles started at a relatively large value then decreased gradually over the next 15–20 min. Particle emission rates for two of the guinea pigs (GP4 and GP6) were only slightly higher at 2 and 3 dpi. The corresponding size distributions, extracted from the final 15 min of each trial (Fig. 4d–f), indicate that the particulates emitted by the anesthetized animals were on average much smaller than those emitted by active guinea pigs in motion (Fig. 3g–i), with a geometric mean diameter of approximately 0.5 µm. Note that the fraction of the smallest particles (between 0.3 and 0.5 µm) for the anesthetized guinea pigs is significantly higher compared to the awake guinea pigs.

**Figure 5:**
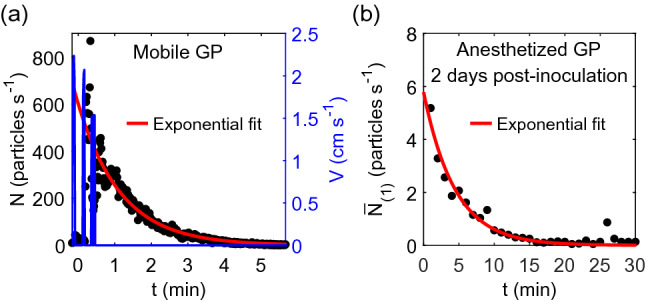
Representative particle emission rate dynamics (**a**) after a guinea pig stops moving in a cage with CC bedding, and (**b**) after placing the aluminum sleeve containing an anesthetized guinea pig inside the measurement cage. In (**a**), the blue lines are the animal velocity. In both (**a**) and (**b**), the red lines are best fits to an exponential decay of the form $N={N}_{0}{e}^{-kt}$ with *k* = 0.85 min^−1^ for (**a**) and 0.22 min^−1^ for (**b**).

It is difficult to interpret the observed decays of particle emission rate in terms of expiratory particles, for two key reasons. First, the particle emission rates from the anesthetized animals decay exponentially, in a manner extremely similar to the decays observed after bursts of motion with the awake mobile guinea pigs. Representative examples for both situations are shown in Fig. 5. Here, we see that the particle rate in either situation is captured well by an exponential decay of the form $N={N}_{0}{e}^{-kt}$. Notably, the time constant $k$ is 0.85 min^–1^ for awake mobile guinea pig (Fig. 5a), and 0.22 min^−1^ for anesthetized guinea pig (Fig. 5b), equivalent to characteristic time scale of ${t}_{c}=\frac{1}{k}=$ 1.18 min, and 4.55 min, respectively. These time scales are of the same order as what one would expect via a standard mass conservation analysis for well mixed air with an initial concentration being diluted by delivery of fresh air. The volume of the cage when empty is $V=$ 26 L, and the flow rate to the APS is $Q=$ 5 L per min, yielding a characteristic time of ${t}_{c}=\frac{V}{Q}\approx$ 5 min. In other words, the observed dynamics are entirely consistent with a rapid aerosolization of large quantities of particulates, followed by an exponential decay as the ventilation slowly removes them. Simultaneously, although the respiratory rate does decline during anesthesia by about 25–33%^34^, there is no physiological reason to expect that the respiratory rate will decline exponentially and result in such a large decrease in particle emission rate as observed here.

The second key reason to interpret the data in terms of dust particulate aerosolization is that our negative control experiments, with humanely euthanized guinea pigs (black points in Fig. 4), yielded extremely similar dynamics as alive, tidally breathing guinea pigs (colored points in Fig. 4). The euthanized animals also exhibited exponential decays, albeit with slightly lower magnitudes, and similar size distributions. Since the animals were not respiring, the particles were necessarily all non-expiratory dust and dander particulates. This observation indicates that despite our best effort to eliminate the background dust particles, simply placing the animal inside the apparatus generated an appreciable amount of dust.

The variability in the long-time particle emission rates (the final 15 min) of Fig. 4a–c complicate efforts to subtract the negative control particle emission rate from the anesthetized animal particle emission rate; at some time points, the anesthetized, respiring animals emitted less particles than the euthanized ones. Even if we ignore this complexity, however, and simply assume that all particles emitted by anesthetized animals are expiratory particles despite the strong evidence to the contrary, they still account for at most 1% of the total particles emitted from an awake, mobile guinea pig. In other words, the data suggest that almost all particles emitted from the guinea pig cage are non-respiratory in origin.

## Discussion

The overarching theme for our results is that a large majority of the particulates emitted from a guinea pig cage appear to be environmental dust, not respiratory droplets. Anesthetized, tidally breathing guinea pigs emitted orders of magnitude fewer particles than were generated by awake, mobile guinea pigs, and, notably, they produced at most only slightly more particles than euthanized guinea pigs under the same conditions. No objects containing fringes consistent with a homogeneous, spherical, liquid droplet larger than 2 µm were observed via the IMI technique, suggesting that, at least in this size range, the airborne particles emitted from guinea pig cages were either solid dust or droplet nuclei of evaporated respiratory droplets. Prior work using IMI with humans focused on speech and coughing^30^, which are well established to yield orders of magnitude more droplets than nasal breathing^35^. To our knowledge there is no clear basis of comparison for how many liquid droplets should be expected from guinea pigs during normal tidal breathing. Nonetheless our data indicate that droplet emission from guinea pigs is rare.

The positive correlations between animal motion and particle counts, observed independently both with the IMI and APS measurements, indicate that animal motion contributes substantially to the generation of airborne particles by guinea pigs. Likewise, the exponential decay in particle concentrations following bursts of animal motion is also most consistent with environmental dust aerosolization. Taken together, the results suggest that almost all of the particles emitted from a cage containing an awake, mobile guinea pig are aerosolized environmental dust rather than expiratory particles.

Although to our knowledge there has been little work characterizing aerosolized dust from individual animal cages like those typically used in airborne disease transmission experiments, at larger scales there has been extensive investigation of dust and particulate matter released from animal processing facilities^36–39^, with some work suggesting that atmospheric wind can carry particulate matter laden with infectious pathogens over distances of many kilometers between different facilities^40,41^. In regard to influenza virus, particularly relevant results were reported by Alonso et al., who measured the concentration, size distribution, and infectivity of particulate matter released from a room containing twelve infected swine; they found that large quantities of micron scale dust was emitted from the room and that viable influenza virus was indeed carried on particulates greater than 2.1 microns in diameter^42^.

Our observations, even in conjunction with these observations in other animal models, do not necessarily indicate that dust particles play any role in influenza virus transmission. Our results do not preclude the possibility that transmission occurs entirely via dried respiratory droplets, and that the large quantities of environmental dust particulates kicked up by animal motion simply confound the measurements. Our prior results, however, confirmed that guinea pigs heavily contaminate their fur and surroundings with influenza virus, and that these contaminated surfaces yield airborne virus capable of infecting susceptible animals in a separate cage^24^.

Influential work by Lowen et al. established that temperature and relative humidity affect the transmissibility of influenza virus between guinea pigs^5–7^; they found that airborne virus transmission occurred readily at cold temperatures (5 °C) but not at hot temperatures (30 °C). This result spurred much investigation of how temperature affects airborne virus survivability, both in animal models and epidemiologically with people^32,43,44^. However, these data can be alternatively explained if the ambient temperature affects the amount of animal motion and the consequent generation of aerosolized fomites. Lowen and colleagues observed qualitatively that guinea pigs appeared lethargic at 30 °C^5^; quantitatively, guinea pigs have been shown to be twice as active at 18 °C than at 30 °C^45^. More recently, experiments by Koster et al. in the ferret model of influenza virus transmission^11^ demonstrated that the total concentration of aerosolized particles transmitted to the susceptible recipient ferret’s cage varied by more than two orders of magnitude during the exposure, ranging from 2 to more than 250 particles/cm^3^. They qualitatively observed that fluctuations in particle transmission rate might relate to observed activity of the donor ferret^11^, albeit without any quantitative measurements of animal activity. The qualitative observation nonetheless suggests that ferrets also produce animal-motion induced spikes similar to those we reported recently with guinea pigs^24^. In combination with prior reports that ferrets contaminate their surrounding dust with virus^46^, these observations raise the possibility that aerosolized fomites play a significant role in the ferret model.

More quantitative details regarding emission from ferrets was provided by Gustin et al., who used an APS to characterize the particles emitted by anesthetized ferrets that were either uninfected or infected with influenza virus^9,12^; however, they did not perform a negative control experiment to quantify non-respiratory particle emissions, such as from euthanized animals. It remains possible that a substantial fraction of the presumed respiratory particles might not actually have been directly exhaled by the ferret, given our results with anesthetized and euthanized guinea pigs (Fig. 4 and Asadi et al.^24^). In a more recent study, Zhou et al.^14^ measured the effect of particle size on the relative efficiency of airborne influenza virus transmission, using impactors to selectively remove particles of a specific size from the air passing between the cages of infected donor and susceptible recipient ferrets. They showed that the transmission efficiency of human influenza declined as the impactor cut-off size was lowered, until no ferret-to-ferret transmission was observed through an impactor that removed airborne particles ≥ 1.5 µm. However, in a separate series of experiments, they confirmed that fine droplet nuclei less than 1.5 μm in size could infect a susceptible recipient ferret when the virus was artificially aerosolized by a nebulizer. These data suggest that influenza virus-infected ferrets do not generate sufficient amounts of fine, virus-laden droplets to transmit infection; rather, ferret-to-ferret transmission of influenza viruses is mediated by airborne particles larger than 1.5 µm. Similarly, Chan et al. recently demonstrated that the transmission of SARS-CoV-2 was reduced but not eliminated by placing a surgical mask barrier between the cages of infected donor and susceptible recipient hamsters. They noted that surgical masks are most efficient at filtering out larger particles “but not the airborne aerosol particles of < 5 µm.”^47^ Importantly, the experimental methods of neither Zhou et al.^14^ nor Chan et al. exclude the possibility that the virus-transmitting airborne particles could have originated from environmental rather than respiratory sources.

Our measurements did not identify the precise source of the aerosolized particulates. The correlation between particle emission rate and guinea pig movement velocity indicates that dust particles are mainly aerosolized from solid sources, but the sources presumably include the bedding, the guinea pig fur and dander, and food and fecal particulates. The size distribution of particles emitted from the guinea pig cage with different types of beddings and also anesthetized guinea pigs shows the effect of different sources on particle size. Considering that particle inhalation and its deposition in respiratory tract is sensitive to particle size^33,48,49^, the size distribution of these particles can affect the probability of disease transmission considerably when using guinea pigs or other animal models to study airborne disease transmission. Furthermore, anesthetized animals do not vocalize, so it is possible that guinea pig vocalization by awake guinea pigs might contribute a larger quantity of expiratory particles, as is observed with vocalizing humans^35,50^. Quantitative measurements of guinea pig vocalization occurrence frequency are necessary to provide rigorous estimates.

As a final comment, if it is correct that aerosolized fomites play a major role in influenza transmission among animal models, then there is a tremendous need to understand how heavily surfaces are contaminated with pathogens and how quickly they are aerosolized, so that this information can be inserted into theoretical models^33,51,52^ of the subsequent spatio-temporal spread of the aerosolized fomites through the air to nearby susceptible animals. The present results motivate more detailed investigation of these issues.

## Conclusions

Our results provide an upper bound for expiratory particle emission rate from a guinea pig cage and clearly indicate that when performing transmission experiments with guinea pigs as animal models, at least 99% of the particles transmitted between two cages, in the size range measured here, may be from sources other than animal’s respiratory tract. Moreover, the results via two independent measurement techniques confirm that particle emission rate from guinea pig cage and guinea pig movement velocity is correlated, and that particle emission rate depends on type of bedding. The data presented here strongly suggests that, when assessing the transmission of respiratory pathogens in animal models, care must be taken to evaluate the relative contributions of expiratory particles and environmental particles and to consider the possibility that transmission occurs at least in part due to aerosolized fomites.

## Supplementary information

Supplementary Information.

## Data Availability

All data is available in the manuscript or the supplementary information. MATLAB codes used for analyzing the data and preparing the figures are available from the corresponding author upon reasonable request.
