# Supplementary material for: Non-respiratory particles emitted by guinea pigs in airborne disease transmission experiments
Source: Sci Rep. 2021 Sep 1;11:17490. doi: 10.1038/s41598-021-96678-w (PMC8410799; doi:10.1038/s41598-021-96678-w)
Supplement: Supplementary file 1 — Supplementary Information. [file 41598_2021_96678_MOESM1_ESM.docx]

**Supplementary Information**

Non-respiratory Particles Emitted by Guinea Pigs in Airborne Disease Transmission Experiments

Sima Asadi, Manilyn J. Tupas, Ramya S. Barre, Anthony S. Wexler,

Nicole M. Bouvier, and William D. Ristenpart

Fig. S1. Photographs of IMI setup.

Fig. S2. Photograph of APS setup.

Table S1. Statistics for power law fit lines shown in Fig. 2c, and Fig. 2d.

Table S2. Statistics for power law fit lines shown in Fig. 3d, Fig. 3e, and Fig. 3f.


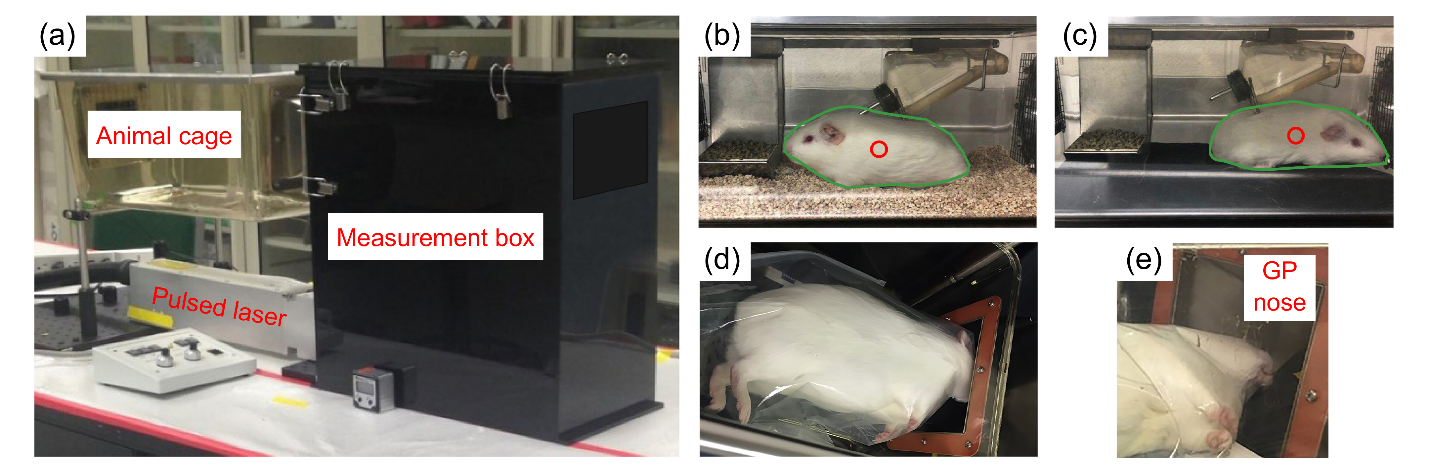


**Fig. S1** **– Photographs of IMI setup.** (a) Photograph of the entire IMI setup. An animal (measurement) cage connected to the black box containing the laser optics and CCD camera. A pulsed laser is used to create a laser sheet inside the box and the particles emitted from the animal cage are carried toward the laser sheet using a small fan. Guinea pig in the cage with (b) CC bedding, and (c) PF bedding. The green line shows the detected guinea pig via standard image analyzing techniques, and the red circle shows the centroid of the detected guinea pig. (d) Anesthetized guinea pig in the cage after placing it in a plastic bag with only its nose exposed (e).


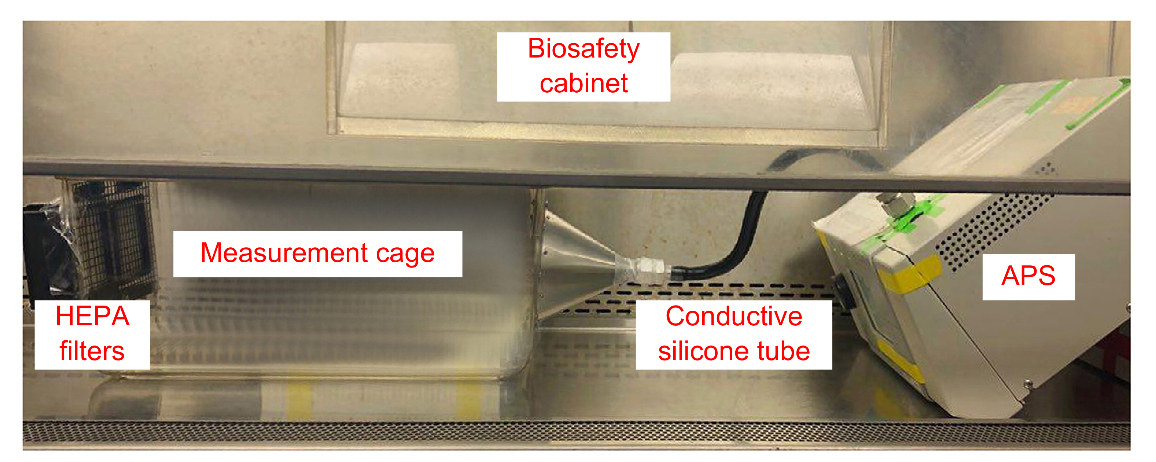


**Fig. S****2 – Photograph of APS setup.** An animal (measurement) cage connected to an APS via a conductive silicone tube placed inside a biosafety cabinet.

**Table S1. Statistics for power law fit lines shown in Fig. 2c, and Fig. 2d.**

|  | GP1 (blue line) | | | GP2 (green line) | | | GP3 (red line) | | | |
| --- | --- | --- | --- | --- | --- | --- | --- | --- | --- | --- |
| Bedding | Exponent | Correlation coefficient | Pearson’s  P-value | Exponent | Correlation coefficient | Pearson’s  P-value | Exponent | Correlation coefficient | Pearson’s  P-value |  |
| CC | 0.92 | 0.91 | 3.3×10^-23^ | 0.82 | 0.68 | 2.0×10^-9^ | 0.84 | 0.92 | 1.4×10^-25^ |  |
| PF | 0.87 | 0.71 | 1.4×10^-10^ | 0.52 | 0.70 | 5.0×10^-10^ | 0.53 | 0.69 | 1.2×10^-9^ |  |

**Table S2. Statistics for power law fit lines shown in Fig. 3d, Fig. 3e, and Fig. 3f.**

|  | GP4 (blue line) | | | GP5 (green line) | | | GP6 (red line) | | | |
| --- | --- | --- | --- | --- | --- | --- | --- | --- | --- | --- |
| Bedding | Exponent | Correlation coefficient | Pearson’s  P-value | Exponent | Correlation coefficient | Pearson’s  P-value | Exponent | Correlation coefficient | Pearson’s  P-value |  |
| CC | 0.93 | 0.80 | 9.6×10^-15^ | 1.36 | 0.57 | 2.5×10^-6^ | 1.52 | 0.65 | 2.2×10^-8^ |  |
| PF | 1.14 | 0.46 | 2.4×10^-4^ | 1.33 | 0.86 | 7.9×10^-19^ | 1.74 | 0.82 | 1.8×10^-15^ |  |
| No | 1.05 | 0.10 | 0.42 | 1.12 | 0.43 | 6.8×10^-4^ | 1.18 | 0.44 | 3.8×10^-4^ |  |
